# Modulatory Effects of Caffeine on Imatinib Binding: A Molecular Docking Study Targeting CYP3A4

**DOI:** 10.3390/life15081247

**Published:** 2025-08-06

**Authors:** Manuel-Ovidiu Amzoiu, Georgeta Sofia Popescu, Emilia Amzoiu, Maria Viorica Ciocîlteu, Costel Valentin Manda, Gabriela Rau, Andrei Gresita, Oana Taisescu

**Affiliations:** 1 Faculty of Pharmacy, University of Medicine and Pharmacy of Craiova, 200638 Craiova, Romania; manuel.amzoiu@umfcv.ro (M.-O.A.); maria.ciocilteu@umfcv.ro (M.V.C.); valentin.manda@umfcv.ro (C.V.M.); gabriela.rau@umfcv.ro (G.R.); 2 Faculty of Food Eng, University of Life Science “King Michael” from Timisoara, 300645 Timisoara, Romania; sofiapopescu@usvt.ro; 3 Department of Physiology, Faculty of Medicine, University of Medicine and Pharmacy of Craiova, 200638 Craiova, Romania; andrei.gresita@umfcv.ro; 4 Faculty of Medicine, University of Medicine and Pharmacy of Craiova, 200638 Craiova, Romania; oana.taisescu@umfcv.ro

**Keywords:** caffeine, imatinib, co-administration, drug interaction, molecular docking, CYP3A4, modulatory effect

## Abstract

Caffeine is a widely consumed psychoactive compound known to influence drug metabolism and efficacy through interactions with key enzymes such as cytochrome P450 3A4 (CYP3A4). This study investigates the molecular impact of caffeine on the binding behavior of imatinib, a first-line BCR-ABL tyrosine kinase inhibitor, using molecular docking simulations. Structural optimization and lipophilicity analyses were conducted using HyperChem, while docking was performed with HEX software (Version 8.0.0) against the CYP3A4 receptor (PDB ID: 1W0E). Two administration scenarios were evaluated: concurrent caffeine–imatinib complex formation and sequential administration with caffeine pre-bound to CYP3A4. The caffeine–imatinib complex exhibited a predicted increase in lipophilicity (logP = 3.09) compared to imatinib alone (logP = −1.29), which may indicate the potential for enhanced membrane permeability and tissue distribution. Docking simulations revealed stronger binding affinity of the complex to CYP3A4 (−350.53 kcal/mol) compared to individual compounds, and improved imatinib binding when CYP3A4 was pre-complexed with caffeine (−294.14 kcal/mol vs. −288.19 kcal/mol). Frontier molecular orbital analysis indicated increased reactivity of the complex (ΔE = 7.74 eV), supporting the hypothesis of altered pharmacodynamic behavior. These findings suggest that caffeine may modulate imatinib’s metabolic profile and therapeutic efficacy by enhancing receptor binding and altering drug distribution. The study underscores the importance of evaluating dietary components during drug development and therapeutic planning, particularly for agents metabolized by CYP3A4.

## 1. Introduction

Caffeine is the most extensively consumed psychoactive compound globally, with widespread intake across diverse populations [1]. Its physiological effects extend beyond immediate central nervous system stimulation, playing a multifaceted role in modulating health outcomes. Pharmacologically, caffeine functions as a non-selective antagonist of adenosine receptors, particularly A1 and A2A subtypes [2], thereby inhibiting the sedative effects typically mediated by endogenous adenosine. This antagonism promotes increased neuronal activity and wakefulness shortly after ingestion. Studies have demonstrated that caffeine enhances alertness, cognitive performance, reaction time, and mood [3]. Beyond this, studies have linked moderate caffeine intake to a reduced risk of certain diseases, including Parkinson’s disease, Alzheimer’s, and some types of cancer [4,5]. For instance, regular coffee drinkers have a significantly lower risk of developing Parkinson’s disease [6,7]. Caffeine also exerts antioxidant effects by scavenging reactive oxygen species, upregulating the expression of endogenous antioxidant enzymes through activation of the nuclear factor erythroid 2–related factor 2 (Nrf2) pathway, and inhibiting lipid peroxidation. Together, these mechanisms contribute to cellular protection against oxidative stress, which may in turn underlie its protective effects against various chronic illnesses [8,9].

However, as with many bioactive compounds, the beneficial effects of caffeine are dose-dependent and are optimally realized when consumed in moderation. Excessive consumption can lead to negative effects, including anxiety, insomnia, increased heart rate, and digestive issues [10,11]. Overuse can also lead to tolerance, where the body becomes desensitized to its effects, leading to the need for higher doses to achieve the same level of alertness [8,12,13]. This can trigger a cycle of dependence, with regular users experiencing withdrawal symptoms such as headaches, irritability, and fatigue when caffeine intake is reduced or stopped [14,15]. Furthermore, in recent years, there has been growing concern regarding the rising use of high-caffeine products, particularly among young people [11]. The rapid growth in the consumption of energy drinks, which often contain far more caffeine than coffee or tea, has raised concerns about misuse and the long-term health risks of excessive caffeine intake [16,17].

Given these concerns, this article examines how caffeine may influence drug efficacy and interactions, with particular attention to the potential risks of high caffeine intake when combined with medications such as imatinib.

Imatinib, a groundbreaking therapeutic agent, has transformed the landscape of cancer treatment [18]. This small molecule has become instrumental in managing a range of malignancies, particularly hematologic cancers such as chronic myeloid leukemia (CML), as well as solid tumors like gastrointestinal stromal tumors (GISTs) [19]. Also known by its trade names Gleevec or Glivec, imatinib was originally developed as a specific inhibitor of the BCR-ABL tyrosine kinase, the oncogenic driver in CML [20]. The discovery of imatinib and its transformative impact on CML treatment stand as a testament to scientific innovation, offering new hope to thousands of patients worldwide. Its FDA approval in 2001 marked a major milestone in oncology as one of the first successful targeted cancer therapies [21,22].

The clinical success of imatinib in CML subsequently facilitated its therapeutic application in other malignancies, most notably gastrointestinal stromal tumors (GISTs), where it functions as a selective inhibitor of the tyrosine-protein kinase KIT, a critical oncogenic driver in these tumors [18]. The precision and selectivity with which imatinib targets these kinases have resulted in exceptional clinical responses, dramatically improving survival rates and outcomes for patients with these cancers. Beyond CML and GISTs, imatinib exemplifies the potential of personalized medicine [21]. Its effectiveness and favorable safety profile have made it a model for the development of other targeted therapies, driving continued research into new agents designed to target specific molecular pathways across various cancer types [19].

Cytochrome P450 3A4 is one of the most important drug-metabolizing enzymes in humans, responsible for the biotransformation of over 50% of pharmaceuticals, including imatinib. Variations in CYP3A4 activity can significantly influence drug efficacy, safety, and clearance rates. Caffeine has been reported to interact with CYP enzymes, potentially altering the metabolism of co-administered drugs. However, few studies have explored how caffeine specifically affects imatinib’s binding to CYP3A4 at the molecular level.

The present study aims to investigate the potential modulatory effects of co-administering imatinib with caffeine-containing foods on imatinib’s binding affinity to CYP3A4 using molecular docking techniques. By this means, we seek to address the methodological challenges and interpretive limitations that arise when evaluating drug–food interactions, while also underscoring the utility of innovative in silico approaches for predicting the impact of dietary components on drug pharmacokinetics and therapeutic efficacy. By examining this often-overlooked dimension of drug development, this work aspires to contribute to a more comprehensive understanding of how dietary factors may influence the clinical success of emerging therapeutic agents.

Molecular docking is a powerful computational technique that has become essential in drug discovery, in biomolecular interaction analysis, and structural biology. It serves as a critical tool for researchers aiming to unravel the intricate interactions between biomolecules at the atomic level [23]. By simulating the binding of small molecules, such as drugs or ligands, to target proteins or nucleic acids, molecular docking offers valuable insights into the mechanisms of action of compounds and their potential therapeutic applications [24]. At its core, molecular docking aims to predict the most favorable orientation and conformation of a ligand within the active site of a target biomolecule. This prediction takes into account steric effects, electrostatic interactions, hydrogen bonding, and hydrophobic forces [25]. Advanced algorithms and scoring functions are used to estimate the binding affinity between the ligand and the target, allowing researchers to identify potential drug candidates, optimize lead compounds, and assess the strength of interactions between ligands and specific biological targets [26]. Beyond its role in drug discovery, molecular docking has a wide range of applications in structural biology, bioinformatics, and chemical biology. Researchers use it to analyze protein–protein interactions and enzyme–substrate complexes and to design novel inhibitors [24,27]. It also plays a key role in understanding the structural basis of diseases, contributing significantly to our knowledge of ligand–receptor interactions. This insight supports the rational design of pharmaceutical agents with improved binding characteristics and therapeutic potential [28,29]. As computational biology and structure-based drug design continue to evolve, molecular docking remains an integral part of the drug discovery process. Ongoing advancements in computational algorithms and hardware are enhancing the accuracy and speed of docking simulations, offering unprecedented opportunities to accelerate the identification and development of novel therapeutics [30].

## 2. Materials and Methods

A chemical modeling study was performed on the molecules of caffeine and imatinib using the HyperChem software program [31]. The 2D molecular structures of the studied compounds were generated using HyperChem software and subsequently optimized through two distinct protocols. The first protocol employed the MM+ (Molecular Mechanics Force Field), while the second utilized the semi-empirical PM3 force field, both implemented in HyperChem (v 8.0.8; HyperCube, Gainesville, FL, USA) [32,33]. Optimization was carried out using the Polak–Ribiere conjugate gradient algorithm, applying an RMS gradient threshold of 0.1 kcal/(Å·mol). The most stable conformations obtained were selected for further analysis.

Additionally, the theoretical logP value, a key descriptor of molecular lipophilicity, was calculated using the QSAR (Quantitative Structure–Activity Relationship) module in HyperChem [31,34]. This parameter offers valuable insights into a compound’s potential interactions with biological membranes and its pharmacokinetic properties. Using the same software, the energies of the frontier molecular orbitals, EHOMO (Highest Occupied Molecular Orbital) and ELUMO (Lowest Unoccupied Molecular Orbital), were also calculated. These parameters were critical in evaluating the electronic structure and reactivity of the studied compounds. The energy difference between HOMO and LUMO orbitals (ΔE = ELUMO − EHOMO) serves as an important indicator of molecular stability. A smaller ΔE value suggests higher chemical reactivity, as the molecule requires less energy to transition between occupied and unoccupied orbitals. Conversely, a larger ΔE indicates greater molecular stability and lower reactivity [34]. These calculations provided insight into how the electronic properties of the molecules may influence their interaction potential and behavior in biological systems.

To explore the binding interactions of these molecules with their respective receptor active sites, docking simulations were conducted using HEX software [33,35]. HEX software was selected for docking studies because it efficiently performs rigid-body docking with combined shape and electrostatic correlation functions. HEX offered advantages for our initial exploratory approach, particularly in handling large, shape-based searches quickly. The software allowed us to perform bidirectional docking: treating one molecule of caffeine as the receptor and one molecule of imatinib as the ligand, and vice versa. We chose this software because it allowed us to save the resulting complexes and use them in subsequent stages of the study. Additionally, HEX provides binding energies as output, which we then used for quantitative comparison between the two scenarios under investigation.

Although HEX software was selected for this study due to its computational efficiency and ability to handle large-scale, shape-based searches, we recognize that it is less commonly employed in molecular docking studies compared to widely used platforms such as AutoDock Vina or Glide. This choice was primarily motivated by the exploratory nature of the current work, which aimed to generate preliminary insights into the potential modulatory effects of caffeine on imatinib binding. We acknowledge, however, that the absence of a formal validation protocol (e.g., re-docking of known ligands, RMSD-based pose comparison, or benchmarking against alternative software) may raise concerns regarding the reproducibility and robustness of the docking outcomes.

Future investigations will incorporate a more rigorous validation framework, including cross-platform comparisons using AutoDock Vina and/or Glide, re-docking of known CYP3A4 ligands to evaluate RMSD values relative to crystallographic poses, and statistical assessment of docking scores across different docking engines. These additional analyses will help to benchmark the reliability of the docking predictions and reduce any potential software-specific bias. Ultimately, future studies that integrate these validation protocols with complementary molecular dynamics simulations and in vitro experiments will be essential for enhancing the robustness and translational relevance of our findings.

A standard docking protocol was applied, utilizing the “Shape + Electro” correlation type to account for both steric and electrostatic complementarity. During the docking process, both imatinib and caffeine were alternately assigned as receptor and ligand to generate a range of possible complex formations. These complexes were then used as ligands in subsequent docking simulations with the cytochrome P450 3A4 (CYP3A4) receptor. To ensure precision in docking calculations, the Hex tool automatically removed all water molecules and other heteroatoms from the input files. It then rotated each protein around its coordinate origin, measuring the separation between the two origins as part of the docking process. For each configuration, a docking score was calculated, and the highest-scoring orientations were selected for further analysis. Following the docking procedure, the top 100 conformations were collected, with the most stable structures, determined by docking scores and structural criteria, chosen for in-depth evaluation. The receptor structures used in this study were obtained from the Protein Data Bank (PDB) [36]. However, we acknowledge that future studies could compare results across different docking platforms to validate findings.

In line with the study objectives, this docking workflow was designed to simulate the potential influence of caffeine on drug binding to a biological receptor under two distinct scenarios: (1) simultaneous administration of caffeine and imatinib, in which a caffeine–imatinib complex generated using HEX was treated as the ligand for docking into the CYP3A4 receptor; and (2) sequential administration, where a protein–caffeine complex was generated and used as the receptor for subsequent imatinib docking. This approach allowed comparative assessment of caffeine’s modulatory effects on imatinib–CYP3A4 binding across different co-administration conditions.

## 3. Results

### 3.1. Physicochemical Properties and Lipophilicity Assessment

To evaluate hydrophobicity, also known as lipophilicity, a key factor in the design of new drugs, we calculated the partition coefficient (logP) values for caffeine and imatinib. These calculations were performed using HyperChem (Table 1).

One crucial factor in understanding drug interactions with biological membranes and protein binding sites is lipophilicity, commonly quantified as the logarithm of the partition coefficient (logP) [37]. This parameter assesses a compound’s affinity for lipid or octanol phases compared to water. LogP values are vital for predicting a molecule’s solubility, permeability, and bioavailability.

In this context, we analyzed the logP values related to the interactions between caffeine and imatinib. The logP values for caffeine, and imatinib highlight the potential for differential interaction profiles with biological receptors when taken concurrently. Caffeine’s presence could influence the solubility or distribution of imatinib, given their similar hydrophilic characteristics. Values of logP as predictors of compound behavior in biological systems were calculated (Table 1) [38]. Understanding these values helps anticipate drug absorption, distribution, and interaction tendencies, which are essential in designing drug regimens, especially when combining compounds with diverse lipophilic and hydrophilic properties. LogP values for the caffeine–imatinib complex, providing insights into how the lipophilicity of the complex differs from that of the individual compounds, were also measured (Table 2).

By examining the logP values of the caffeine–imatinib complex, we can better understand how caffeine may alter the distribution, solubility, and overall pharmacokinetic behavior of imatinib in a biological setting. The caffeine–imatinib complex exhibits a logP of 3.09, which is substantially higher than the logP of imatinib alone (−1.29). This marked increase in lipophilicity suggests an enhanced affinity for lipid-rich environments, such as cellular membranes. Greater lipophilicity may facilitate improved membrane permeability and tissue retention, potentially affecting imatinib’s pharmacokinetics by promoting its accumulation in lipid-rich compartments and prolonging its systemic presence. Conversely, this shift toward a more lipophilic profile could imply reduced solubility in aqueous environments, which may influence drug distribution and metabolism. Collectively, these logP values highlight the significant impact that caffeine binding can exert on imatinib’s pharmacokinetic behavior. The observed increase in lipophilicity for the caffeine–imatinib complex suggests a greater potential for enhanced tissue permeability and retention, which could prolong the drug’s activity in lipid-rich tissues.

It is important to clarify that the observed increase in lipophilicity (logP) of the caffeine–imatinib complex is a theoretical in silico prediction derived from computational modeling. Real-world pharmacokinetic outcomes, including changes in absorption, distribution, metabolism, and excretion, would require experimental validation in in vitro and in vivo conditions.

Lipophilicity plays a critical role in determining the pharmacokinetic behavior of drug candidates, particularly their absorption and metabolism. Highly lipophilic compounds generally exhibit enhanced passive diffusion across biological membranes such as the intestinal epithelium, which facilitates oral absorption [39,40]. However, lipophilic drugs also tend to have higher affinity for cytochrome P450 isoforms, including CYP3A4, potentially leading to increased first-pass metabolism and reduced systemic availability [41]. This dual role highlights the need for an optimal balance in lipophilicity to achieve efficient absorption without excessive metabolic clearance.

In the context of the present study, the predicted increase in lipophilicity of the caffeine–imatinib complex may suggest altered pharmacokinetic properties, but any such effects remain speculative. Further experimental studies, such as permeability assays, microsomal stability testing, and clinical pharmacokinetic evaluations, will be necessary to confirm whether the complexation of imatinib with caffeine meaningfully alters its pharmacokinetic profile. These findings underscore the importance of accounting for caffeine’s potential influence on drug disposition, as complex formation with caffeine may modify the pharmacokinetic and pharmacodynamic profiles of medications by altering their relative affinities for lipid-rich and aqueous biological environments.

Lipophilicity values (logP) were calculated using the QSAR module in HyperChem, which applies fragmental methods based on octanol–water partition coefficients. Typical logP values for drugs range from approximately −5 (very hydrophilic) to +5 (very lipophilic), with most orally active drugs falling between −1 and +3.

The caffeine–imatinib complex was generated using HEX software. Caffeine and imatinib were individually energy-minimized in HyperChem and subsequently subjected to docking in HEX, where caffeine was treated as the receptor and imatinib as the ligand to model non-covalent interactions. The resulting complex geometry was then exported and treated as a single ligand for subsequent docking to CYP3A4.

**Figure 1:**
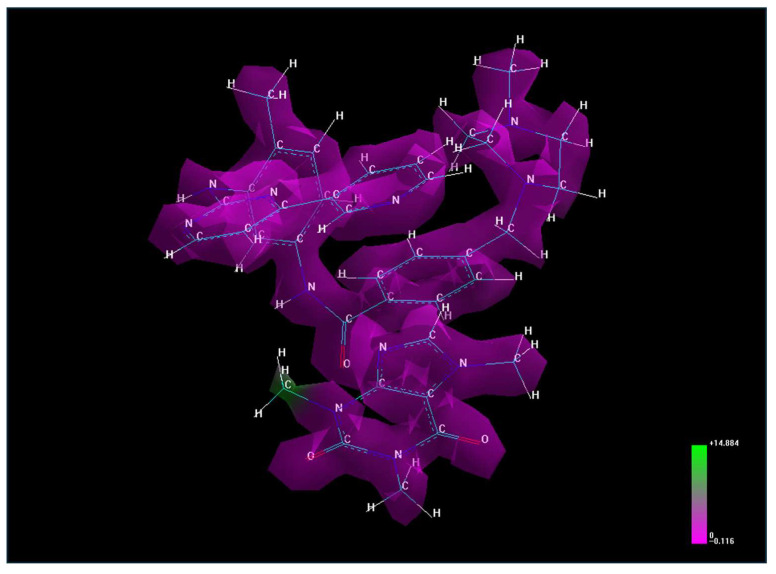
The electrostatic potential for caffeine–imatinib complex.

Molecular lipophilicity can also be assessed through electrostatic potential, a valuable property for analyzing and predicting reactive molecular behavior. This potential highlights the polarity of specific regions on the molecule’s van der Waals surface, visualized as a three-dimensional electron density map with electrostatic potential values represented by color (Figure 1). Areas with high potential values (positive zones, shown in green) strongly attract water molecules, while areas with lower values (negative zones, shown in purple) do not exhibit an attractive character, making them hydrophobic regions.

The electrostatic potential map of the caffeine–imatinib complex reveals a predominance of non-polar regions with limited polar, water-attracting functional groups, consistent with the observed increase in lipophilicity (logP = 3.09).

### 3.2. Frontier Molecular Orbital Analysis and Reactivity

The frontier orbitals are critically important for a molecule, just as the valence shell is essential in the electronic structure of an atom. In molecular frontier orbital theory, the Highest Occupied Molecular Orbital (HOMO) and the Lowest Unoccupied Molecular Orbital (LUMO) play a central role in determining how a molecule interacts with other species. The HOMO reflects the molecule’s ability to donate electrons, while the LUMO represents its capacity to accept electrons. Their energy levels and spatial distribution provide essential insight into a compound’s reactivity, stability, and role in intermolecular interactions, making them a fundamental concept in computational chemistry, drug design, and molecular docking studies.

The ligand (drug)–biological receptor interaction can be effectively analyzed using frontier molecular orbitals (FMOs), where the HOMO and LUMO energy levels offer insights into the electronic reactivity and binding potential of the studied compounds (Table 3).

The EHOMO value provides insight into a molecule’s donor properties or its tendency to undergo oxidation. Conversely, the ELUMO value allows for an assessment of a molecule’s acceptor properties or its tendency to undergo reduction. Molecules with high EHOMO energy are most susceptible to electrophilic attack, while those with maximum ELUMO values are more likely to undergo nucleophilic attack.

The energy difference between the HOMO and LUMO molecular levels (ΔE = ELUMO − EHOMO) is also a significant chemical parameter, as it indicates molecular stability; a low value suggests high reactivity [42]. Based on the values presented in Table 3, caffeine is the most stable molecule (ΔE = 8.4093), while the most reactive combination is the caffeine–imatinib complex (ΔE = 7.7398). Additionally, comparing the ΔE values of imatinib (ΔE = 7.8556) and the caffeine–imatinib complex (ΔE = 7.7398) reveals a lower ΔE for the complex, indicating increased reactivity of imatinib upon complexation. This aligns with the observation that this complex has improved bioavailability and distribution compared to the uncomplexed compound.

### 3.3. Molecular Docking with CYP3A4: Binding Affinity and Structural Insights

In the subsequent phase of the study, molecular docking simulations were performed to evaluate the interaction between the caffeine–imatinib complex and the cytochrome P450 3A4 (CYP3A4) enzyme, utilizing crystallographic data from the Protein Data Bank (PDB ID: 1W0E) [43]. CYP3A4 is essential for drug metabolism, influencing the breakdown and efficacy of various compounds [44]. By integrating structural data, our aim is to reveal how these drugs and their complexes interact with CYP3A4, offering insights into the three-dimensional characteristics of these interactions and their potential pharmacological implications.

The binding energy values reflect the relative stability and affinity of each compound or complex within molecular interactions, with more negative values indicating stronger and more stable binding. These data provide insights into the potential behavior of caffeine and imatinib—individually or in combination—within biological systems, particularly regarding their interaction dynamics and binding efficiencies (Table 4).

It is important to note that larger ligands often produce more negative docking scores because of increased steric and electrostatic contacts with the receptor. Therefore, these energy values should be interpreted qualitatively and not as absolute binding affinity indicators. The caffeine–imatinib complex (−350.53) demonstrates a significant reduction in energy compared to imatinib alone, suggesting an intermediate level of stability when imatinib is complexed with caffeine. This energy reduction indicates that caffeine interaction may alter the structural stability or binding efficiency of imatinib, likely affecting how the drug is distributed and metabolized. The moderate stability of this complex could mean that caffeine has an impact on imatinib’s pharmacodynamics, potentially reducing its efficacy or altering its binding with specific targets. Imatinib (−288.19) shows a relatively high (less negative) energy value, which could signify a lower stability or binding affinity in its isolated form. This aligns with imatinib’s more hydrophilic profile, which may result in a different binding behavior in biological environments. The less stable energy profile could influence how imatinib interacts with receptors or enzymes, impacting its therapeutic profile and absorption rate compared to more lipophilic compounds. Caffeine (−179.09) has the highest energy value, indicating the least stability and binding potential among the compounds listed. This aligns with its hydrophilic nature, suggesting a lower affinity for lipid-rich environments and reduced binding potential within biological targets. Caffeine’s low binding energy reflects its relatively rapid absorption and elimination in the body, as it is less likely to form stable, long-lasting complexes with proteins or other compounds. The differences in energy values between individual compounds and their caffeine complexes suggest that caffeine may influence the pharmacokinetic behavior of imatinib [45]. Imatinib’s energy shift when complexed with caffeine is more pronounced, indicating that caffeine could significantly impact its stability, potentially altering its therapeutic profile [46].

Overall, these energy values highlight that caffeine interaction may reduce the stability of imatinib, potentially affecting its pharmacokinetic properties, distribution, and binding efficiency. This could lead to altered therapeutic effects or interactions when imatinib is co-administered with caffeine. It is important to note that docking energy values are influenced by the size and surface area of the ligand. Larger molecules may produce lower (more negative) energies due to increased steric and electrostatic contacts, and these values should not be interpreted as direct measures of binding affinity without further normalization or experimental validation

Furthermore, binding interactions between imatinib and the CYP3A4 enzyme, both in the absence and presence of caffeine, reveal how co-administration may influence drug affinity and stability. Comparative energy values indicate that caffeine slightly enhances imatinib’s binding to CYP3A4, potentially modifying its metabolic rate and therapeutic persistence. More negative binding energies correspond to stronger interactions, suggesting that caffeine could modulate the pharmacological behavior of imatinib (Table 5).

For the CYP3A4 + caffeine + imatinib complex (−294.14 kcal/mol), the presence of caffeine resulted in a modest reduction in binding energy compared to imatinib binding alone (–288.19 kcal/mol). This difference suggests that caffeine may slightly enhance the stability of the CYP3A4–imatinib interaction, potentially by inducing subtle conformational adjustments in CYP3A4 that favor imatinib binding. Enhanced binding affinity could, in turn, influence imatinib’s metabolic rate and prolong its active presence in systemic circulation. By contrast, the CYP3A4 + imatinib configuration (−288.19 kcal/mol) exhibited a comparatively weaker interaction, consistent with a lower intrinsic affinity for CYP3A4 in the absence of caffeine, which may affect the drug’s metabolic processing by the enzyme. The presence of caffeine enhances imatinib’s binding affinity to some extent, potentially leading to minor changes in its stability or interaction dynamics. These findings suggest that caffeine may play a role in modulating imatinib’s binding to CYP3A4, which could have implications for its therapeutic profile. For imatinib, caffeine’s effect is more moderate, slightly enhancing the stability of the CYP3A4–imatinib complex. This suggests a potential, but less pronounced, effect on imatinib’s metabolism, which could result in a minor extension of its action or a slight alteration in its clearance rate. Overall, these findings suggest that caffeine could alter the pharmacokinetics of drugs metabolized by CYP3A4 by affecting their binding affinities to the enzyme. This could have clinical implications, as co-administration with caffeine may influence the therapeutic duration or efficacy of drugs like imatinib.

**Figure 2:**
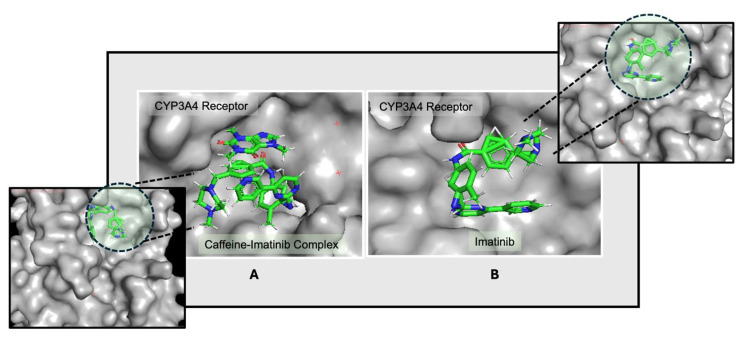
Docking conformations of the CYP3A4 receptor with (**A**) the caffeine–imatinib complex and (**B**) imatinib, highlighting differences in orientation and binding affinity. The ligand molecules are represented as sticks colored according to the atoms present (C—green, N—blue, O—red, H—white), and the receptor is highlighted in light gray. The differences in the ligand pockets are visible between the two configurations [47].

Further binding site analysis reveals distinct interaction patterns for the drug–ligand complexes, with binding energies expressed as negative values, where more negative values indicate stronger and more stable interactions. These energy profiles highlight the relative binding affinities of each drug configuration and offer structural insight into their stability and potential efficacy (Figure 2).

The data suggest that imatinib may respond differently in the presence of caffeine, as caffeine appears to induce a shift toward an alternative binding site where the caffeine–imatinib complex demonstrates a modestly stronger interaction. This possible enhancement in binding affinity indicates that caffeine could influence imatinib’s interaction dynamics, which may in turn impact its pharmacokinetic and pharmacodynamic properties.

**Figure 3:**
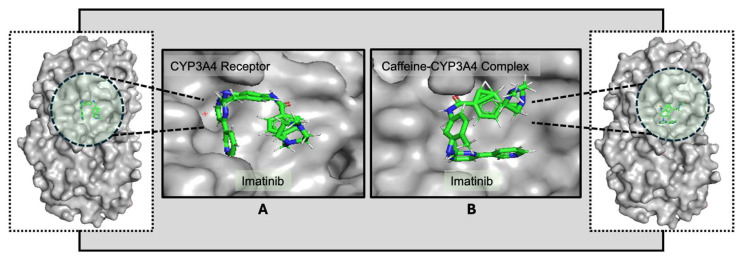
Docking conformations of the receptor CYP3A4 with imatinib (**A**) and of the caffeine–CYP3A4 complex with imatinib (**B**), highlighting differences in orientation. The ligand molecules are represented as sticks colored according to the atoms present (C—green, N—blue, O—red, H—white), and the receptor is highlighted in light gray. The altered conformation in (**B**) suggests that caffeine may modulate imatinib’s interaction with CYP3A4 [48].

Further studies, particularly three-dimensional structural analyses with the CYP3A4 receptor, are necessary to deepen our understanding of the mechanisms and potential clinical implications of these binding interactions. The influence of caffeine on drug binding is evident in the interaction patterns observed with the CYP3A4 receptor. Despite targeting different binding sites, the presence of caffeine enhances binding strength, indicating increased stability in the drug–ligand interactions and suggesting potential modulation of pharmacokinetics and efficacy (Figure 3). The data indicate that both drugs may exhibit a comparable response to the CYP3A4 structure in the presence of caffeine, potentially showing enhanced binding affinity [47]. This increased affinity in the presence of caffeine could potentially impact the pharmacokinetics and pharmacodynamics of these drugs.

The observed differences in binding energies may indicate that caffeine could influence these interactions. When co-administered with imatinib, caffeine appears to modestly increase its binding at the target site. Similarly, pre-exposure of CYP3A4 to caffeine was associated with a slight further increase in binding strength, which could reflect a potential modulation of drug–enzyme interactions depending on the timing of administration. A clearer understanding of how imatinib interacts with CYP3A4 in the presence of caffeine will be important for informing optimal drug intake strategies, minimizing possible drug–food interactions, and anticipating pharmacokinetic and pharmacodynamic effects. Additional structural analyses, particularly in the context of the CYP3A4 receptor, will be required to delineate these binding sites in greater detail and to clarify their potential clinical implications.

## 4. Discussion

This study provides molecular-level insights into the potential modulatory effects of caffeine on the pharmacokinetics and binding affinity of imatinib using computational docking methodologies. The marked increase in lipophilicity (logP = 3.09) observed in the caffeine–imatinib complex compared to imatinib alone (logP = −1.29) indicates a significant shift toward enhanced membrane permeability and potential tissue retention. This physicochemical transformation may have clinical implications, suggesting prolonged systemic exposure and altered biodistribution of imatinib when co-administered with caffeine.

The frontier molecular orbital analysis further supports this hypothesis. The reduced HOMO–LUMO energy gap (ΔE = 7.7398 eV) in the complex indicates a higher reactivity and possibly greater interaction potential with biological targets, aligning with enhanced docking scores. This suggests that caffeine complexation could sensitize imatinib to enzyme-mediated interactions, particularly those involving CYP3A4. Molecular docking against CYP3A4 (PDB ID: 1W0E) revealed that the caffeine–imatinib complex exhibits a significantly stronger binding affinity (−350.53 kcal/mol) than either imatinib (−288.19 kcal/mol) or caffeine alone (−179.09 kcal/mol). Interestingly, even when caffeine was pre-bound to the receptor, imatinib’s binding was modestly enhanced (−294.14 kcal/mol), implying that caffeine may induce a conformational shift in CYP3A4 that favors imatinib docking.

These findings raise important considerations for drug–diet interactions, particularly for drugs extensively metabolized by CYP3A4 [49], such as imatinib, midazolam, cyclosporine, and certain statins. Caffeine-induced changes in CYP3A4 binding may influence the pharmacokinetics or efficacy of these agents.

While the caffeine-induced enhancement of binding affinity may prolong drug action, it also raises the possibility of altered clearance or unintended pharmacodynamic effects, particularly in subjects with high dietary caffeine intake [50,51]. This is consistent with the literature indicating that dietary components, such as grapefruit juice or polyphenols, can modulate cytochrome P450 activity [49,52]. However, the extent of such effects remains uncertain and requires experimental validation.

To contextualize these findings, it is valuable to compare them with the existing literature on caffeine’s interaction with CYP3A4 substrates. For instance, Sevrioukova (2023) [44] provided structural evidence that caffeine can bind simultaneously at multiple sites within the CYP3A4 active site, potentially influencing the binding of co-substrates and modulating enzymatic activity. This observation supports our finding that caffeine may induce a conformational change in CYP3A4, enhancing imatinib’s docking affinity within the enzyme’s active site [44]. In a separate study, Cameron et al. demonstrated cooperative binding of caffeine and acetaminophen within CYP3A4, emphasizing how caffeine can alter the binding topology and affinity of concurrent ligands. This phenomenon parallels the dual-binding scenario modeled in our study, wherein caffeine either precedes or co-binds with imatinib, resulting in different binding strengths [53]. These studies align with our computational predictions, reinforcing the clinical importance of investigating food–drug interactions at the molecular level, particularly in CYP-mediated metabolic pathways.

Notably, the limitations of this study must be acknowledged. First, the simulations performed assumed idealized, static receptor–ligand interactions and did not account for the dynamic temporal and metabolic processes that occur in vivo. The use of HEX software, while appropriate for the exploratory nature of this study, represents a methodological limitation because it is less extensively validated in the molecular docking community compared to widely used software platforms such as AutoDock Vina or Glide. This factor, coupled with the absence of a formal validation protocol, may affect the robustness and reproducibility of the docking results.

Additionally, this study relies exclusively on static docking simulations, which provide a ‘snapshot’ of ligand–receptor interactions at a single point in time. Such static models do not capture the dynamic conformational flexibility of proteins or the temporal stability of the ligand–receptor complexes. Molecular dynamics simulations would enable the exploration of these time-dependent properties, providing a more comprehensive understanding of the binding interactions, including potential conformational rearrangements of CYP3A4 and the ligands.

Future research will therefore incorporate cross-platform docking validation, MD simulations, and experimental approaches such as in vitro enzyme assays or microsomal stability studies. These complementary methods will allow us to assess not only the reliability of the predicted docking poses but also their biological relevance, ultimately improving the translational significance of the findings.

Second, the binding affinities derived from docking simulations cannot be directly equated to pharmacological efficacy or safety. Such computational predictions must be complemented by in vitro or in vivo validation to confirm their biological relevance. The exploratory nature of the present study is further underscored by the absence of experimental validation, which limits the extent to which definitive conclusions can be drawn. Nevertheless, the combined use of lipophilicity analysis, frontier molecular orbital calculations, and docking simulations provides a robust in silico framework for generating hypotheses about potential drug–caffeine interactions.

It is also important to interpret the docking scores with caution. The more negative binding energy values observed for the caffeine–imatinib complex may in part reflect its larger molecular surface area relative to imatinib alone, resulting in increased steric and electrostatic contacts with the receptor. Docking scores should therefore not be viewed as absolute measures of binding affinity, as they may not correlate linearly with true biological binding strength.

Finally, the clinical implications of these computational findings remain speculative. Enhanced binding of imatinib to CYP3A4 in the presence of caffeine could theoretically reduce the drug’s metabolic clearance and prolong systemic exposure. While this may potentially improve therapeutic efficacy, it could also elevate the risk of adverse effects, particularly at higher doses or in patients with reduced drug clearance capacity. As such, any dietary recommendations concerning caffeine intake in patients receiving imatinib should be made with caution and substantiated by rigorous clinical studies.

In conclusion, while the absence of molecular dynamics simulations and experimental validation constitutes a major limitation, this study provides a valuable preliminary framework. It offers testable hypotheses and lays a foundation for future in vitro, in vivo, and dynamic simulation studies aimed at elucidating the pharmacokinetic and pharmacodynamic consequences of dietary caffeine on drugs metabolized by CYP3A4.

Lastly, from a clinical perspective, the predicted modulation of CYP3A4 binding affinity by caffeine could theoretically influence imatinib metabolism, potentially altering systemic exposure, therapeutic efficacy, and the risk of adverse effects. CYP3A4 is a major metabolic enzyme responsible for the biotransformation of more than 50% of prescribed drugs, and dietary components have been shown to significantly affect its activity. Although there are currently no documented clinical cases specifically linking caffeine intake to altered imatinib efficacy or safety, previous studies have demonstrated that caffeine can impact the metabolism of other CYP3A4 substrates, suggesting the plausibility of a similar interaction. These findings underscore the importance of considering dietary habits, particularly caffeine consumption, during therapeutic planning for patients receiving imatinib. Nevertheless, we emphasize that our results are hypothesis-generating, and further in vitro, in vivo, and clinical pharmacokinetic investigations will be required to determine whether caffeine co-administration has a clinically meaningful impact on imatinib therapy.

## 5. Conclusions

In conclusion, this study suggests that caffeine, through direct molecular interactions, can modulate both the bioavailability and binding behavior of imatinib. These effects appear to vary depending on whether caffeine is consumed concurrently with imatinib or prior to its administration, underscoring the importance of administration timing when dietary stimulants are present. By influencing drug-binding sites, caffeine may impact both the pharmacokinetics and pharmacodynamics of imatinib. Although caffeine appears to enhance binding affinity, it may also alter the dynamic characteristics of drug–receptor interactions. These findings suggest that co-administration strategies could potentially be optimized to improve therapeutic outcomes; however, such approaches should be guided by further experimental and clinical validation.

These findings provide valuable insights into drug–food interactions, particularly concerning widely consumed dietary components like caffeine. Understanding such interactions is essential for optimizing dosing regimens and improving therapeutic efficacy, reinforcing the need for further research into the structural and temporal dimensions of co-administration. Patient dietary habits are, therefore, a critical consideration in maximizing drug efficacy. Diet can significantly influence drug absorption, distribution, metabolism, and excretion, with certain foods and beverages either enhancing or diminishing therapeutic effectiveness. For instance, caffeine has been shown to modulate drug metabolism by affecting enzyme activity, which can lead to increased potency or accelerated clearance. Moreover, broader dietary patterns may further modulate pharmacokinetic and pharmacodynamic processes, highlighting the need for a more integrated approach to pharmacotherapy. Incorporating individual nutritional habits into treatment planning can enhance the principles of personalized medicine, leading to more informed prescribing decisions and improved therapeutic outcomes. Ultimately, integrating dietary guidance with drug therapy may be essential for achieving the full therapeutic potential of many medications.

## Figures and Tables

**Table 1:** Structure and partition coefficient of the studied compounds.

Structure	Compound	logP (Octanol/Water)
Caffeine	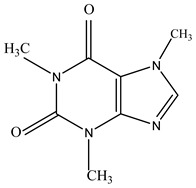	−1.06
Imatinib	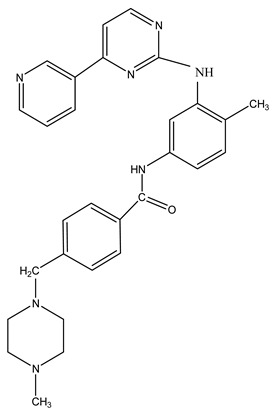	−1.29

**Table 2:** Partition coefficients of the studied compounds.

Compound	logP (Octanol/Water)
Caffeine–imatinib	3.09
Imatinib–caffeine	3.09

**Table 3:** Energy values for the studied compounds.

COMPOUND	E_HOMO_, eV	E_LUMO_, eV	ΔE, eV
Caffeine	−8.8969	−0.4876	8.4093
Imatinib	−9.0245	−1.1689	7.8556
Caffeine–imatinib	−8.9326	−1.1928	7.7398

HOMO—Highest Occupied Molecular Orbital, LUMO—Lowest Unoccupied Molecular Orbital, ΔE = ELUMO − EHOMO.

**Table 4:** Binding energy values with the CYP3A4 receptor [42].

Compound	Energy [kcal/mol]
Caffeine–imatinib	−350.53
Imatinib	−288.19
Caffeine	−179.09

**Table 5:** Binding energy values of the drug with the CYP3A4 and CYP3A4 + caffeine receptor [33].

Receptor	Ligand	Energy [kcal/mol]
CYP3A4 + caffeine	Imatinib	−294.14
CYP3A4	Imatinib	−288.19

## Data Availability

The data presented in this study are available on request from the corresponding authors.
